# Phytoplankton trigger the production of cryptic metabolites in the marine actinobacterium *Salinispora tropica*

**DOI:** 10.1111/1751-7915.13722

**Published:** 2020-12-05

**Authors:** Audam Chhun, Despoina Sousoni, Maria del Mar Aguiló‐Ferretjans, Lijiang Song, Christophe Corre, Joseph A. Christie‐Oleza

**Affiliations:** ^1^ School of Life Sciences University of Warwick Coventry UK; ^2^ University of the Balearic Islands Palma Spain; ^3^ Department of Chemistry University of Warwick Coventry UK; ^4^ IMEDEA (CSIC‐UIB) Esporles Spain

## Abstract

Filamentous members of the phylum Actinobacteria are a remarkable source of natural products with pharmaceutical potential. The discovery of novel molecules from these organisms is, however, hindered because most of the biosynthetic gene clusters (BGCs) encoding these secondary metabolites are cryptic or silent and are referred to as orphan BGCs. While co‐culture has proven to be a promising approach to unlock the biosynthetic potential of many microorganisms by activating the expression of these orphan BGCs, it still remains an underexplored technique. The marine actinobacterium *Salinispora tropica*, for instance, produces valuable compounds such as the anti‐cancer molecule salinosporamide but half of its putative BGCs are still orphan. Although previous studies have used marine heterotrophs to induce orphan BGCs in *Salinispora*, its co‐culture with marine phototrophs has yet to be investigated. Following the observation of an antimicrobial activity against a range of phytoplankton by *S. tropica*, we here report that the photosynthate released by photosynthetic primary producers influences its biosynthetic capacities with production of cryptic molecules and the activation of orphan BGCs. Our work, using an approach combining metabolomics and proteomics, pioneers the use of phototrophs as a promising strategy to accelerate the discovery of novel natural products from marine actinobacteria.

## Graphical abstract

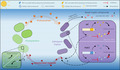

## Introduction

Soil actinomycetes are a rich source of drug‐like natural products, to which we owe up to 70% of all microbial antibiotics used today (Bérdy, 2012). Identification of novel secondary metabolites from this extensively studied phylum has, however, stalled over the last few decades as a result of the recurring rediscovery of already known compounds. This has led in recent years to a thriving interest for the study of new microorganisms, with the rational that ecologically distinct microorganisms produce equally distinct secondary metabolites (Molinski *et* *al*., 2009; Wilson and Brimble, 2009; Bull and Goodfellow, 2019). For instance, the heterotrophic bacteria *Salinispora* drew particular attention when discovered, as it was the first obligate marine actinomycete described (Jensen *et al*., 1991; Mincer *et al*., 2002; Jensen and Mafnas, 2006) and has since proven to be an important source of new natural products for the pharmaceutical industry (Feling *et al*., 2003; Maldonado *et al*., 2005; Buchanan *et al*., 2005; Asolkar *et al*., 2010). Despite the increasing number of novel strains identified with promising biosynthetic capacities, many hurdles in natural product discovery remain. Most of these microbial secondary metabolites are encoded by groups of colocalized genes, called biosynthetic gene clusters (BGCs), which are now more easily identified because of the improvement in sequencing technologies and bioinformatic tools (Medema *et al*., 2011). The majority of these discovered BGCs, however, have yet to be linked to their products and are called orphan BGCs. They are generally considered to be either silent ‐ because of a low level of expression or inactivation of their biosynthetic genes ‐ or the metabolites they produce are cryptic ‐ difficult to detect and isolate (Reen *et al*., 2015; Rutledge and Challis, 2015). The observation of numerous orphan BGCs in genome‐sequenced microorganisms has resulted in a growing interest in developing biological or chemical means to activate such clusters (Abdelmohsen *et al*., 2015; Onaka, 2017). One of the simplest and most efficient methods described in the literature relies on co‐cultivation of different microbes to elicit novel natural product biosynthesis (Slattery *et al*., 2001; Bertrand *et al*., 2014; Wakefield *et al*., 2017).

The genome of the marine actinobacterium *Salinispora tropica* comprises at least 20 putative BGCs of which 11 are orphan (Table 1; Udwary *et al*., 2007; Penn *et al*., 2009). Recent studies have shown that some *Salinispora* strains co‐inoculated with various marine heterotrophs could produce one or several antimicrobial compounds, which remain uncharacterized as traditional analytical chemistry methods did not allow their identification and no candidate BGC was proposed (Patin *et al*., 2016; Patin *et al*., 2018). While co‐culturing appears to be a promising mean to activate orphan BGCs in *Salinispora*, it remains an underexplored technique to unravel the biosynthetic potential of members of this genus. Additionally, little has been done to establish the BGCs that are activated under such culturing conditions. Meanwhile, ‐omic technologies have become instrumental in the exploitation of marine microbes for biotechnological applications and more particularly for the discovery of novel natural products (Hartmann *et al*., 2014; Palazzotto and Weber, 2018). Combining metabolomics with proteomics analyses has indeed proven successful in uncovering new secondary metabolites and in linking those to their corresponding orphan BGCs in several *Streptomyces* species, but has not yet been applied to the genus *Salinispora* (Schley *et al*., 2006; Bumpus *et al*., 2009; Chen *et al*., 2013; Gubbens *et al*., 2014; Owens *et al*., 2014; Du and Wezel, 2018).

**Table 1:** Biosynthetic gene clusters of *Salinispora tropica* CNB‐440.

BGC name	Biosynthetic class	Product	Genetic location (strop_)	Size (kb)	References
*sal*	Polyketide/non‐ribosomal peptide	Salinosporamide	RS05130‐RS05275	41.8	Feling *et al*. (2003)
*lom*	Polyketide	Lomaiviticin	RS10930‐RS11215	62.2	Kersten *et al*. (2013)
*des*	Hydroxamate	Desferrioxamine	RS12775‐RS12855	19.2	Roberts *et al*. (2012)
*spo*	Polyketide	Sporolide	RS13560‐RS13730	49.2	Dineshkumar *et al*. (2014)
*slm*	Polyketide	Salinilactam	RS13850‐RS13965	82.0	Udwary *et al*. (2007)
*lym*	Polyketide/non‐ribosomal peptide	Lymphostin	RS15295‐RS15350	25.0	Miyanaga *et al*. (2011)
*terp1*	Terpenoid	Sioxanthin	RS16250‐RS16295	10.4	Richter *et al*. (2015)
*spt*	butyrolactone	Salinipostin	RS20900‐RS20940	11.1	Amos *et al*. (2017)
*terp2*	Terpenoid	Sioxanthin	RS22405‐RS22445	11.7	Richter *et al*. (2015)
***pks1***	**Polyketide**	**NA**	**RS02980‐RS03095**	**30.9**	**NA**
***nrps1***	**Non‐ribosomal peptide**	**NA**	**RS03375‐RS03535**	**37.5**	**NA**
***amc***	**Carbohydrate**	**NA**	**RS11765‐RS11795**	**6.6**	**NA**
***bac1***	**Ribosomal peptide**	**NA**	**RS11800‐RS12275**	**19.2**	**NA**
***pks3***	**Polyketide**	**NA**	**RS12510‐RS12630**	**23.3**	**NA**
***sid2***	**Non‐ribosomal peptide**	**NA**	**RS13260‐RS13385**	**40.7**	**NA**
***sid3***	**Non‐ribosomal peptide**	**NA**	**RS13985‐RS14120**	**29.2**	**NA**
***sid4***	**Non‐ribosomal peptide**	**NA**	**RS14125‐RS14260**	**40.8**	**NA**
***bac2***	**Ribosomal peptide**	**NA**	**RS14265‐RS15290**	**19.0**	**NA**
***pks4***	**Polyketide**	**NA**	**RS21120‐RS21540**	**10.0**	**NA**
***nrps2***	**Non‐ribosomal peptide**	**NA**	**RS22250‐RS22350**	**34.7**	**NA**

Characterized and orphan (in bold) BGCs of *S. tropica* CNB‐440.

Here we report the discovery of cryptic secondary metabolites produced by *S*.* tropica* CNB‐440. By using an approach combining metabolomics and proteomics, we investigated how marine microbial phototrophs, and their photosynthate, induce the production of new metabolites and activate the expression of orphan BGCs in *S*.* tropica*. This strategy confirms microbial interactions as a promising and simple approach for future discovery of novel natural products.

## Results

### *Salinispora tropica* has antimicrobial activity on a diverse range of marine phototrophs

**Fig. 1:**
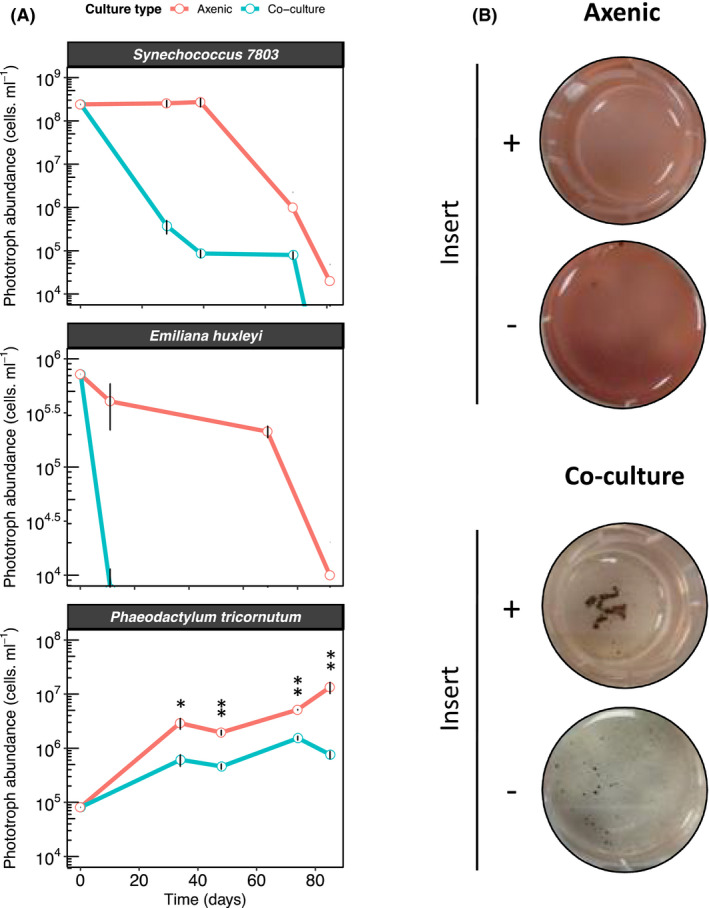
*Salinispora tropica* inhibits the growth of marine phototrophs *via* the secretion of an antimicrobial molecule (A) *S. tropica* inhibits marine phototrophs growth in co‐culture. Cultures of three marine phototrophs grown axenically (red lines) and in co‐culture with *Salinispora tropica* (blue lines). Graph shows mean ± standard deviation of three biological replicates. Statistically significant cell abundances are indicated (T‐test, significant * at *P*‐value < 0.05 and ** at *P*‐value < 0.01). B. *Synechococcus* growth inhibition by *S. tropica* mediated by a diffusible molecule. The cyanobacterium was grown axenically and in co‐culture with *S. tropica,* either separated with a 0.4 µm pore membrane insert (+) or without separation (‐). Photographs of representative cultures of three biological replicates are shown, 7 days after inoculation. Red pigmentation is characteristic of healthy *Synechococcus* cells, while cell bleaching indicates cell death.

Unlike other heterotrophs, which usually enhance the growth of phototrophic organisms when in co‐culture (e.g. Christie‐Oleza *et al*., 2017; Sher *et al*., 2011), *S*.* tropica* showed a clear antimicrobial activity on marine phytoplankton (Fig. 1A). All three phototrophic model species tested, namely the cyanobacteria *Synechococcus* sp. WH7803, the coccolithophore *Emiliania huxleyi* and the diatom *Phaeodactylum tricornutum*, showed a strong decline in the presence of *S*.* tropica*, being especially remarkable for the two former strains (Fig. 1A). While also affected, the diatom *P*.* tricornutum* was not killed by *S*.* tropica* but, instead, its cells densities were significantly maintained one order of magnitude lower than when incubated axenically. While we were able to monitor the inhibition of the phototroph, we were unable to assess the growth profile of *S*.* tropica* in the co‐culture due to its growth in dense cell aggregates preventing traditional monitoring techniques (e.g. optical density, colony‐forming unit or flow cytometry).

We were therefore interested in characterizing the nature of this inhibition. While members of other *Salinispora* species, such as *Salinispora arenicola*, are known to biosynthesize antibiotic molecules (Asolkar *et al*., 2010), no antimicrobial compound has yet been characterized in *S*.* tropica* CNB‐440. Previous studies have shown, however, that *S*.* tropica* is able to outcompete other heterotrophs in co‐culture by secreting siderophores leading to iron depletion (Patin *et al*., 2016). To evaluate whether iron sequestration could explain the negative interactions observed in the present phototroph‐*Salinispora* system, we supplemented the co‐cultures with increasing concentrations of iron (Fig. S1). The results obtained suggest that the antimicrobial phenotype was not due to siderophore activity, as saturating amounts of iron could not rescue the growth of the phototrophs.

We then hypothesized that a yet unknown antimicrobial compound, to which our photosynthetic microorganisms are sensitive to, could be produced by *S*.* tropica*. To test this assumption, we set up co‐cultures in which the *S*.* tropica* and *Synechococcus* strains were physically separated by a porous filter, preventing direct cell to cell interactions while allowing the diffusion of small molecules (Fig. 1B). *S*.* tropica* was still able to impair *Synechococcus* proliferation in these experimental conditions, confirming that a secreted molecule was causing the death of the phototroph.

### Phototrophs elicit the production of novel cryptic metabolites in *S*.* tropica*

**Fig. 2:**
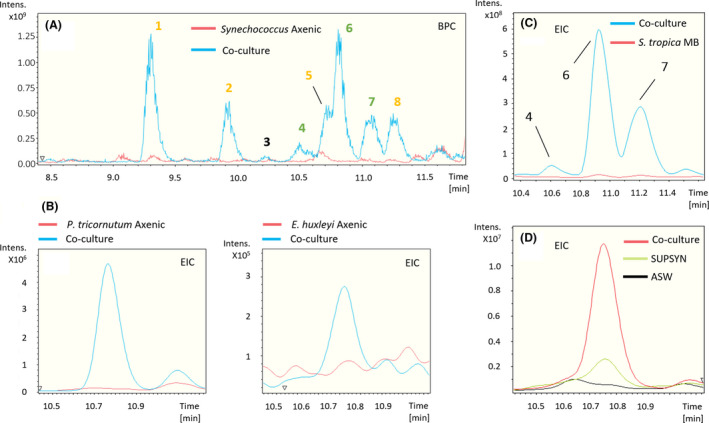
Marine phototrophs trigger the production of cryptic molecules in *S. tropica* (A) *S. tropica* produces detectable small molecules in co‐culture with *Synechococcus*. Overlaid base peak chromatograms (BPCs) of *Synechococcus* culture concentrated supernatants, when grown for 35 days in artificial sea water (ASW) either axenically (red) or in co‐culture with *S. tropica* (blue). Peaks characteristic of the co‐culture condition are labelled from 1 to 8. Colour of the labels indicate groups of related compounds. B. Other marine phototrophs also trigger the production of metabolite 6 by *S. tropica* as observed in panel A. Figure shows extracted ion chromatograms for the molecule 6 EIC 435.2 ± 0.1 in the supernatants of the phototrophs (*P. tricornutum*, left panel; *E. huxleyi*, right panel) grown axenically (red) and in co‐culture with *S. tropica* (blue). C. The production of the related molecules 4, 6 and 7 is dependent on the presence of photosynthate rather than high‐nutrient availability. Graph shows extracted ion chromatograms for all three cryptic molecules EIC (464.2; 435.2; 449.2) ± 0.5 in the concentrated supernatants of *S. tropica* grown axenically for 35 days in marine broth (*S. tropica* MB, red) or in co‐culture with *Synechococcus* in ASW (Co‐culture, blue). D. Cryptic molecule production is triggered by nutrients released by *Synechococcus* rather than cell to cell interactions. Graph shows extracted ion chromatograms for the cryptic molecule 6 (EIC 435.2 ± 0.5) in the supernatant of *S. tropica* grown axenically for 14 days either in artificial sea water (ASW, black line) or in a conditioned *Synechococcus* supernatant (SUPSYN, green line); and in co‐culture with *Synechococcus* (Co‐culture, red line). SUPSYN is equivalent to the ‘*Synechococcus* Axenic’ condition in panel A; cryptic metabolites were only detected after *S. tropica* incubation.

We analysed the co‐culture supernatants using non‐targeted metabolomics to identify the pool of secondary metabolites secreted by *S*.* tropica* in response to the different phototrophs. The *Synechococcus‐S*.* tropica* co‐culture revealed eight molecular ions that were not present in the respective axenic cultures (Fig. 2A). These molecules were further characterized by high‐resolution MS/MS analysis, from which we generated empirical chemical formulae, allowing us to assign most of them to two subgroups of related compounds being: (i) ions **1**, **2**, **5** and **8**; and (ii) ions **4**, **6** and **7** (Fig. 2A; Table S1).

Ions **1**, **2**, **5** and **8** were derivatives of salinosporamide; a well‐characterized molecule produced by *S*.* tropica* that presents a unique fused γ‐lactam‐β‐lactone bicyclic ring structure (Feling *et al*., 2003), and that is now being tested as a drug because of its anti‐cancer properties. Molecules **5** and **8** are consistent with known degradation products of salinosporamide A and B, respectively (Denora *et al*., 2007; Fig. S2), while molecules **1** and **2** are proposed to result from the nucleophilic addition of Tris (the buffering agent used in the ASW culture medium) to the lactone ring of salinosporamide A and B, respectively (Fig. S2). These salinosporamide sub‐products were further confirmed by their absence when (i) Tris was not added (Fig. S3), or (ii) salinosporamide mutants that no longer produced these metabolites, *that is* salA^‐^ and salL^‐^ (Eustáquio *et al*., 2009), were used (Fig. S4). In order to test the activity of salinosporamide and its derivatives on the phototrophs, we co‐cultured *Synechococcus* with both salinosporamide mutants. Salinosporamide and its derivatives were not responsible for the antimicrobial activity as both deficient mutants were still able to inhibit the phototroph (Fig. S5).

The second group of ions, *that is* peaks **4**, **6** and **7**, were also related. Molecule **6** gave a *m/z* value of 435.2609 [M + H]^+^; based on the accuracy of this value and the isotopic pattern the empirical chemical formula C_22_H_35_N_4_O_5_ was predicted by the DataAnalysis software (Table 2). The predicted formula for molecule **4** suggests that, with a 28.9900 Da mass difference when compared to **6**, the compound had lost one hydrogen and gained an atom of nitrogen and oxygen. MS/MS analyses confirmed that both molecules **4** and **6** had an identical molecular fragment (i.e. *m/z* 276.1600 ± 0.0001 [M + H]^+^, with the empirical chemical formula C_16_H_22_NO_3_), indicating that the two molecules share a core backbone (Table 2; Fig. S6, S7). Similarly, molecule **7** had the same chemical formula as **6** but with the addition of a methyl group (14.0155 Da mass difference; Table 2). Finally, MS/MS fragmentation of the three molecules **4**, **6** and **7** resulted in the related molecular fragments *m/z* 171.088, 142.0979 and 156.1135, respectively, which showed differences in masses and empirical formulae identical to that observed between their corresponding parent ions (Table 2; Fig. S6, S7). Molecule **3** did not share an obvious link to any other metabolites and, therefore, was considered a new biosynthesized product of *S*.*tropica* (Table 2). Most interestingly, the search for compounds with the same molecular formulae as **3**, **4**, **6** or **7** in multiple databases (e.g. Reaxys, SciFinder, Dictionary of NP) returned no known natural product, suggesting that they are novel compounds. Unfortunately, despite multiple attempts, the isolation of these molecules has so far proven too challenging for their structural elucidation.

**Table 2:** Characteristics of the cryptic molecules. MS Peak numbering is based on HPLC retention time.

MS Peak	Observed *m/z*	Chemical formulae for [M + H]^+^ (calculated *m/z*; err [ppm])	Observed *m/z* (MS/MS)	Chemical formulae for [M + H]^+^ (calculated *m/z*; err [ppm])
3	438.1701	[C_28_ H_24_ N O_4_]^+^ (438.1700; −0.3)	194.0817	[C_10_H_12_NO_3_]^+^ (194.0812; −2.6)
			177.1279	[C_12_H_17_O]^+^ (177.1274; −2.8)
**4**	464.2509	[C_22_ H_34_ N_5_ O_6_]^+^ (464.2504; −1.2)	276.1600	[C_16_H_22_NO_3_]^+^ (276.1594; −2.2)
			171.0880	[C_6_H_11_N_4_O_2_]^+^ (171.0877; −2.1)
			154.0615	[C_6_H_8_N_3_O_2_]^+^ (154.0611; −2.7)
**6**	435.2609	[C_22_ H_35_ N_4_ O_5_]^+^ (435.2602; −1.7)	372.2290	[C_21_H_30_N_3_O_3_]^+^ (372.2282; −2.1)
			276.1599	[C_16_H_22_NO_3_]^+^ (276.1594; −1.6)
			142.0979	[C_6_H_12_NO_3_]^+^ (142.0975; −2.7)
**7**	449.2764	[C_23_ H_37_ N_4_ O_5_]^+^ (449.2758; −1.3)	156.1135	*ND*

High‐resolution LC‐(+)ESI‐MS *m/z* values and predicted chemical formulae for [M + H]^+^ are provided. *ND* indicates ions for which DataAnalysis was unable to generate chemical formulae.

The production of these novel compounds was only triggered by the presence of the phototrophs as they were only detected in the co‐cultures of all three phototrophs (Fig. 2A and B), but not when grown in mono culture – as shown by the absence of these metabolites when *S*.* tropica* was grown alone in mineral ASW or nutrient‐rich media MB (Fig. 2C and D). Furthermore, we confirmed that the supernatant of a phototroph culture – containing the photosynthate – was enough to induce such metabolite production (Fig. 2D).

### Photosynthate triggers the expression of orphan gene clusters in *S*.* tropica*

Having detected novel secondary metabolites produced by *S*.* tropica* in response to phototroph‐released photosynthate, we set out to investigate how it affected the induction of its BGCs. To this end, we analysed and compared the proteome of *S*.* tropica* when grown in presence of the phytoplankton photosynthate – *that is* in conditioned *Synechococcus* supernatant (SUPSYN) where the cryptic molecules are produced – and in nutrient‐rich broth – *that is* marine broth (MB) where the cryptic molecules are not detected. Considering that the medium SUPSYN is effectively artificial seawater with some phototroph‐released nutrients, we also incubated *S*.* tropica* in fresh artificial seawater (ASW) as a control. The rationale behind using the SUPSYN medium rather than co‐inoculating *S*.* tropica* with *Synechococcus* was twofold. First, it had the advantage to avoid *Synechococcus* proteins that may interfere with the correct detection of *Salinispora* proteins. Second, we reasoned that it would reduce potential variations in protein expression caused by *Synechococcus*‐*Salinispora* cell to cell interactions and/or the limitation in carbon availability that would occur early in the co‐culture in ASW.

**Fig. 3:**
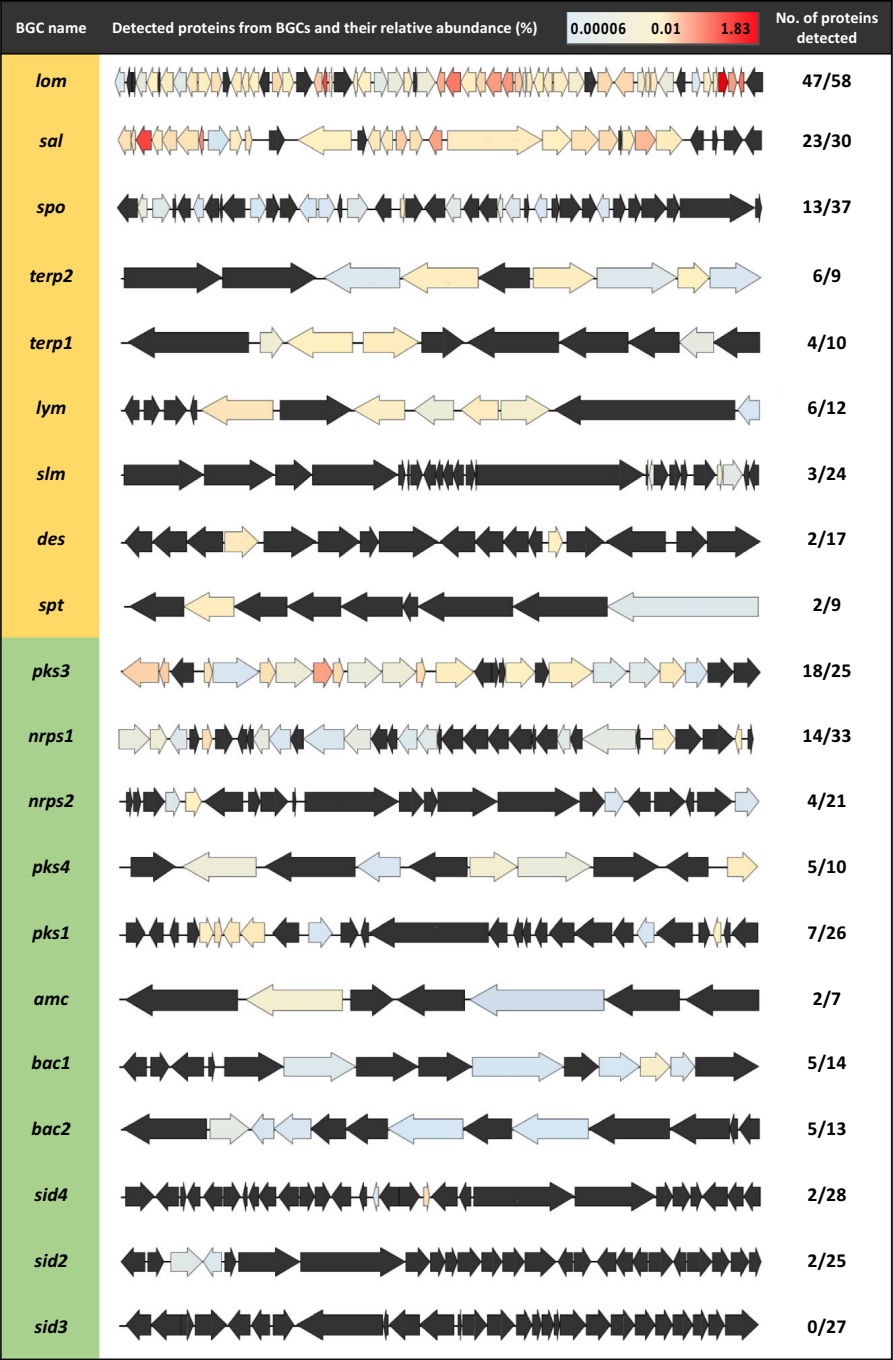
Photosynthate allows the expression of orphan biosynthetic gene clusters in *S. tropica*. Characterized (orange) and orphan BGCs (green) in *S. tropica* CNB‐440 detected by high‐throughput proteomics when grown with photosynthate (SUPSYN) for 5 days. Genes are coloured according to the relative abundance of their corresponding proteins. Those that were not detected are represented in black.

Overall, the datasets obtained contain close to 1,900 proteins (i.e. 1869, 1797 and 1831 detected proteins for the MB, ASW and SUPSYN conditions, respectively) representing about 40 % of the total expected proteome of *S*.* tropica* (Table S2; File S1). We found that 10 % of those detected proteins could be linked to BGCs (Table S2), a figure consistent with the estimated percentage of the *S*.* tropica* genome to be devoted to natural product assembly (Udwary *et al*., 2007). Interestingly, while the actual numbers of BGC‐related proteins were similar in all three conditions tested, we found that their cumulative abundances accounted for a significantly higher proportion of the proteomes when cells were grown in presence of the phytoplankton’s photosynthate compared to both MB and ASW (Table S2). As we believed this observation suggests that phototrophic cells prompt an increase of *S*.* tropica* secondary metabolism, we were interested in identifying which BGCs were expressed and influenced by photosynthate. Surprisingly, we were able to detect proteins encoded by almost all of *S*.* tropica*’s BGCs regardless of the incubation medium, including 10 of its 11 orphans BGCs (Fig. 3, File S1).

Of particular interest were the orphan BGCs *pks3* and *nrps1*, for which we detected 72% (18/25) and 42% (14/33) of their encoded proteins, respectively (Fig. 3). Moreover, the *pks3* BGC was noticeably highly detected as eight of its detected proteins showed a relative abundance above 0.1% (Fig. 3; Table S3). While it has been previously suggested that *pks3* may produce a spore pigment polyketide, very little experimental evidence is available in the literature, and the product of *pks3* had not been confirmed (Kersten *et al*., 2013). On the other hand, the non‐ribosomal peptide synthetase (NRPS) gene cluster *nrps1* has only been predicted to produce a non‐ribosomal dipeptide (Penn *et al*., 2009). Intriguingly, the most abundant proteins detected from this *nrps1* BGC were the non‐ribosomal peptide synthetase (A4X2Q0), an adenylation domain‐containing protein (A4X2R4), a condensation domain‐containing protein (A4X2R5) and an ATP‐dependent Clp protease subunit (A4X2S2), with a relative abundance of 0.004%, 0.004%, 0.001% and 0.121% respectively (Table 3). While the three former are thought to direct the biosynthesis of the non‐ribosomal peptide, the later may be involved in conferring resistance to the synthesized antimicrobial compound (Kirstein *et al*., 2009), as further discussed below.

**Table 3:** Detected proteins from the *nrps1* orphan BGC in *S. tropica* CNB‐440 grown with photosynthate.

Protein ID	*Homologue* (% identity/% similarity) *Organism* [Protein ID]	Putative function	Relative abundance (%; *n* = 3)
A4X2P5	*ridA* (33/54) *Bacillus subtilis* [P37552.3]	2‐iminobutanoate/2‐iminopropanoate deaminase	0.014
A4X2P8	MFS transporter (28/41) *Mycobacterium tuberculosis* [A0A0H3M5L9.1]	MFS transporter	0.018
A4X2Q0	*srfAB* (28/45) *Bacillus subtilis* [Q04747.3]	Non‐Ribosomal Peptide Synthetase^a^ [C‐A‐PCP]	0.004
A4X2Q2	*fabG* (34/56) *Vibrio harveyi* [P55336.1]	Ketoreductase domain^a^	0.002
A4X2R0	*fadE25* (24/40) *Mycrobacterium leprae* [P73574.1]	Acyl‐CoA dehydrogenase	0.003
A4X2R1	*YdiO* (28/42) *Escherichia coli K‐12* [p0A9U8.1]	Acyl‐CoA dehydrogenase	0.002
A4X2R4	*ProA* (gramicidin S synthase) (32/47) *Brevibacillus brevis* [P0C064.2]	Adenylation domain^a^	0.004
A4X2R5	*AlaA* (gramicidin synthase subunit B) (26/45) *Brevibacillus parabrevis* [Q70LM6.1]	Condensation domain‐containing protein^a^ [C‐PCP‐TE]	0.001
A4X2R7	*argG* (53/73) *Nitratiruptor* sp. [A6Q3P9.1]	Argininosuccinate synthase	0.001
A4X2R8	*Fmt* (32/48) *Stenotrophomonas maltophilia* [B2FIR3.1]	Methionyl‐tRNA formyltransferase	0.004
A4X2S2	*clpP* (69/85) *Frankia casuarinae* [Q2J9A8.1]	ATP‐dependent Clp protease proteolytic subunit^b^	**0.121**
A4X2S4	*MjK1* (25/41) *Methanocaldococcus jannaschii* [Q57604.1]	Potassium channel protein	0.002
A4X2S5	*korB* (65/77) *Mycobacterium tuberculosis* [O53181.1]	2‐oxoglutarate oxidorectudase subunit beta	0.006
A4X2S6	*korA* (66/78) *Mycobacterium tuberculosis* [O53182.3]	2‐oxoglutarate oxidoreductase subunit alpha	0.004

^a^ Core biosynthetic enzyme.

^b^ Protein potentially involved in self‐resistance to a proteasome inhibitor.

The already characterized *lom* and *sal* BGCs were also abundantly detected with 81% (47/58) and 77% (23/30) of their encoded proteins detected, respectively, some representing high‐relative abundances within the proteome (Fig. 3). The BGC *lom* is linked to the cytotoxic glycoside lomaiviticin molecule (Kersten *et al*., 2013). However, this metabolite previously showed no antimicrobial activity on co‐cultured heterotrophic organisms (Patin *et al*., 2018) and, hence, it is unlikely to cause the antimicrobial phenotype observed on the phototrophs in this study. The high abundance of the *sal* cluster, producing the salinosporamide compound, is not surprising given the high detection of this metabolite by LC‐MS (Fig. 2A).

**Fig. 4:**
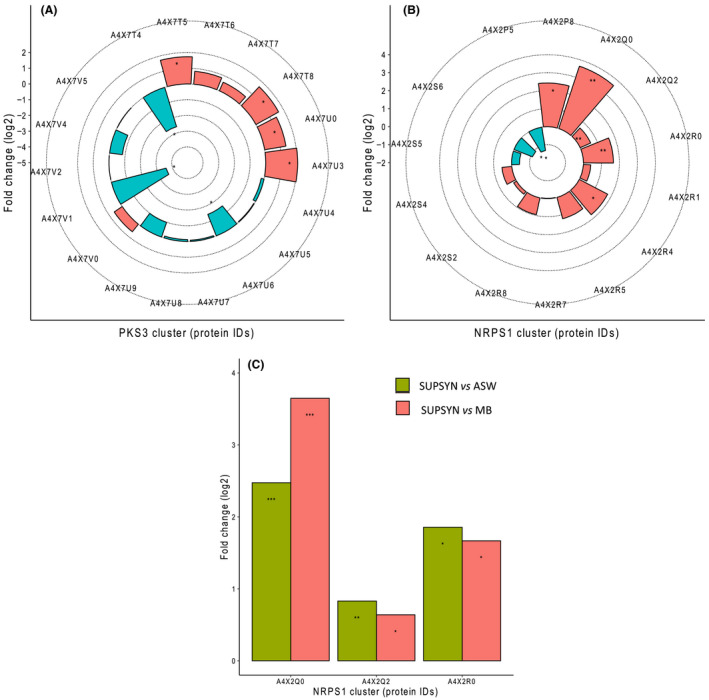
Photosynthate increases the expression of the orphan *nrps1* BGC in *S. tropica*. Photosynthate and other nutrients influence several proteins from the orphan BGCs *pks3* (A) and *nrps1* (B), in comparison with cells grown in MB. Up‐ (red) and down‐regulated (blue) proteins in the presence of the photosynthate (SUPSYN) are shown. Statistically significant fold changes are indicated by an asterisk (T‐test, significant at *q*‐value < 0.05). Enzymes further analysed for a *post hoc* test are indicated by a double asterisk. C. Photosynthate specifically triggers an up‐regulation of several proteins from the *nrps1* BGC. Up‐regulated proteins in the presence of photosynthate compared to ASW (green) and MB (red) are shown. These proteins showed no significant difference between the MB and ASW conditions. Tukey HSD test, * significant at *q*‐value < 0.05; ** significant at *q‐*value < 0.01; *** significant at *q*‐value < 0.001.

Interestingly, although all BGCs appeared to be similarly active between conditions with regards to the detection of their proteins (i.e. the same proteins were generally present in the different proteomes, rather than being conditional to one growth medium), a thorough comparative analysis of their expression levels revealed that phototroph‐released nutrients could specifically trigger several proteins within the BGC *nrps1* (Fig. 4). For instance, the orphan *pks3* BGC showed an up‐regulation of pivotal enzymes for polyketide biosynthesis when cells were grown in the SUPSYN medium compared to MB. Namely, an acyl‐CoA ligase (A4X7T8), a 3‐ketoacyl‐ACP synthase (A4X7U0) and a long‐chain fatty acid‐CoA ligase (A4X7U3) were up‐regulated 3.1, 2.6 and 4.1‐fold, respectively (Fig. 4A). These variations in protein detection could not, however, be confidently attributed solely to the presence of photosynthate because they were also observed at similar levels in the ASW condition. This could indicate that the orphan *pks3* cluster’s expression is regulated by nutrient availability as it is less active in a nutrient‐rich broth like MB. On the other hand, the activity of the BGC *nrps1* was more clearly up‐regulated by the presence of photosynthate (Fig. 4B and C). This included three core biosynthetic enzymes, namely a non‐ribosomal peptide synthetase (12.5‐fold change increase in the SUPSYN condition compared to the MB treatment; A4X2Q0), a ketoreductase domain‐containing protein (1.6‐fold change; A4X2Q2) and an adenylation domain‐containing protein (3.4‐fold change; A4X2R4). Unlike the *pks3* BGC, photosynthate did specifically trigger an increase in the expression of the BGC *nrps1*, as it exhibited a similar up‐regulation in the SUPSYN condition compared to the two other treatments, *that is* ASW and MB (Fig. 4C).

## Discussion

We show that *S*.* tropica* is able to inhibit the growth of both marine cyanobacteria and eukaryotic phototrophs by some, yet, unidentified mechanism (Fig. 1). This observation broadens the potential role and impact that members of the *Salinispora* genus have on marine microbial communities. Members of the three *Salinispora* species are widely distributed bacteria found in all tropical and subtropical oceans (Mincer *et al*., 2002; Bauermeister *et al*., 2018). While mostly inhabiting marine sediments, bacteria from this genus have also been isolated from the microbiota of seaweeds, marine sponges and more recently corals (Jensen *et al*., 2005; Asolkar *et al*., 2010; Ocampo‐Alvarez *et al*., 2020). In marine sponges, it is suggested they influence the sponge microbiota through the production of acyl homoserine lactone molecules and antibiotics (Singh *et al*., 2014; Bose *et al*., 2017). Similarly, different species of *Salinispora* were shown to possess distinct mechanisms to outcompete co‐occurring marine heterotrophs in sediments, *that is* through the production of siderophores to deplete iron or antimicrobial molecules (Patin *et al*., 2017; Tuttle *et al*., 2019), although no antimicrobial compound has yet been identified for *S*. *tropica* (Patin *et al*., 2018). We herein provide the first evidence that *Salinispora* might not only directly influence heterotrophic communities, but also kill both prokaryotic and eukaryotic phytoplankton to which they are exposed, *for example* when these sediment out of the water column (Guidi *et al*., 2016), grow in sunlit coastal sediments (Patin *et al*., 2017) or are part of the same coral‐associated microbiota (Ocampo‐Alvarez *et al*, 2020).

In *Salinispora*, co‐culture has been employed but failed so far to result in the discovery of novel natural products from the genus. Patin and colleagues showed that *S*.*arenicola* and *S*.* tropica* strains exhibited antimicrobial activities against various marine heterotrophs (Patin *et al*., 2016, 2017, 2018). Although some of these interactions were found to be caused by already known bioactive compounds (e.g. siderophores), several of those were mediated by an uncharacterized mechanism and overall no new natural product was identified (Patin *et al*., 2016). In a following manuscript, the interactions between *S*.* tropica* CNY‐681 and several heterotrophs were further explored by MS/MS networking analysis (Patin *et al*., 2018). The networks generated revealed unknown molecular families unique to the co‐cultures but they were either (i) not proven to be produced by *S*.* tropica* rather than the co‐inoculated heterotroph or (ii) related to the already characterized siderophore desferrioxamine E (Patin *et al*., 2018). Using metabolomics, we here characterized the production of cryptic metabolites in the type strain *S*.* tropica* CNB‐440^T^ in response to the presence of a range of prokaryotic and eukaryotic phytoplankton species. Molecules with identical *m/z* were never reported in the literature and they were also distinct from the molecular families described by Patin et al. when *S*.* tropica* was grown with marine heterotrophs (Patin *et al*., 2018). This finding confirms the overlooked potential of co‐culturing *Salinispora* specifically with phytoplankton to elicit the production of novel secondary metabolites.

While we were successful in identifying and obtaining the molecular formulae of the cryptic metabolites produced in response to phytoplanktonic photosynthate (Fig. 2, Table 2), we were unable to isolate and identify the compound responsible for the antimicrobial effect on the marine phototrophs by using traditional bioactivity‐guided assays with HPLC fractionation of crude extracts (data not shown). This mechanism proved similarly elusive in previous studies, where *S*.* tropica* showed an antimicrobial activity on marine heterotrophs, but the molecule responsible was not identified (Patin *et al*., 2016; Patin *et al*., 2018). The parallelism between our observations and those described in the literature suggests that the active compound(s) may be the same. We reason that the compound’s instability, and/or synergic effect of several molecules required for activity, could explain the difficulty in identifying the antimicrobial agent. For instance, the large number of structurally related metabolites resulting from the chemical reaction of salinosporamide with various compounds (i.e. water as shown in Denora *et al*., 2007; and Tris in this study) may support this hypothesis, as the antimicrobial molecule may be similarly unstable. The diversity of products arising from a single BGC may also be due to the promiscuity of the biosynthetic enzymes utilizing structurally related primary precursors. This results in a range of compounds, each produced at lower titres than a single natural product, and ultimately hamper the isolation of sufficient amounts of the compounds of interest. Whatever the case, we show that *S*.* tropica* can produce a broad‐range antibiotic able to affect both unicellular prokaryotes and eukaryotes alike, such as the marine diatom and coccolithophore tested in our study. Such a broad‐range antimicrobial could suggest a mode of action affecting a common target present in both types of cells such as the proteasome, a proteolytic complex present in the three domains of life (Becker and Darwin, 2016).

Exploring the proteome of *S*.* tropica* exposed to photosynthate, we detected proteins encoded by almost all its BGCs, including most of its orphan BGCs (Fig. 3). Notably, the *sal* BGC, producing the salinosporamide compound, was one of the most highly expressed BGC as most of its proteins were detected with high‐relative abundance. This finding is in agreement with previous studies that have shown by transcriptomics that the BGC *sal* is highly and constitutively expressed when grown in nutrient‐rich A1 medium (Amos *et al*., 2017). Also, the high expression of this BGC correlated with a noticeable detection of salinosporamide derivatives by LC‐MS. Therefore, the abundant detection of several orphans BGC proteins, including those from *pks3* and *nrps1* BGCs, may be promising candidates responsible for the biosynthesis of the cryptic metabolites detected by LC‐MS and, potentially, the antimicrobial activity observed on co‐cultured phototrophs.

**Fig. 5:**
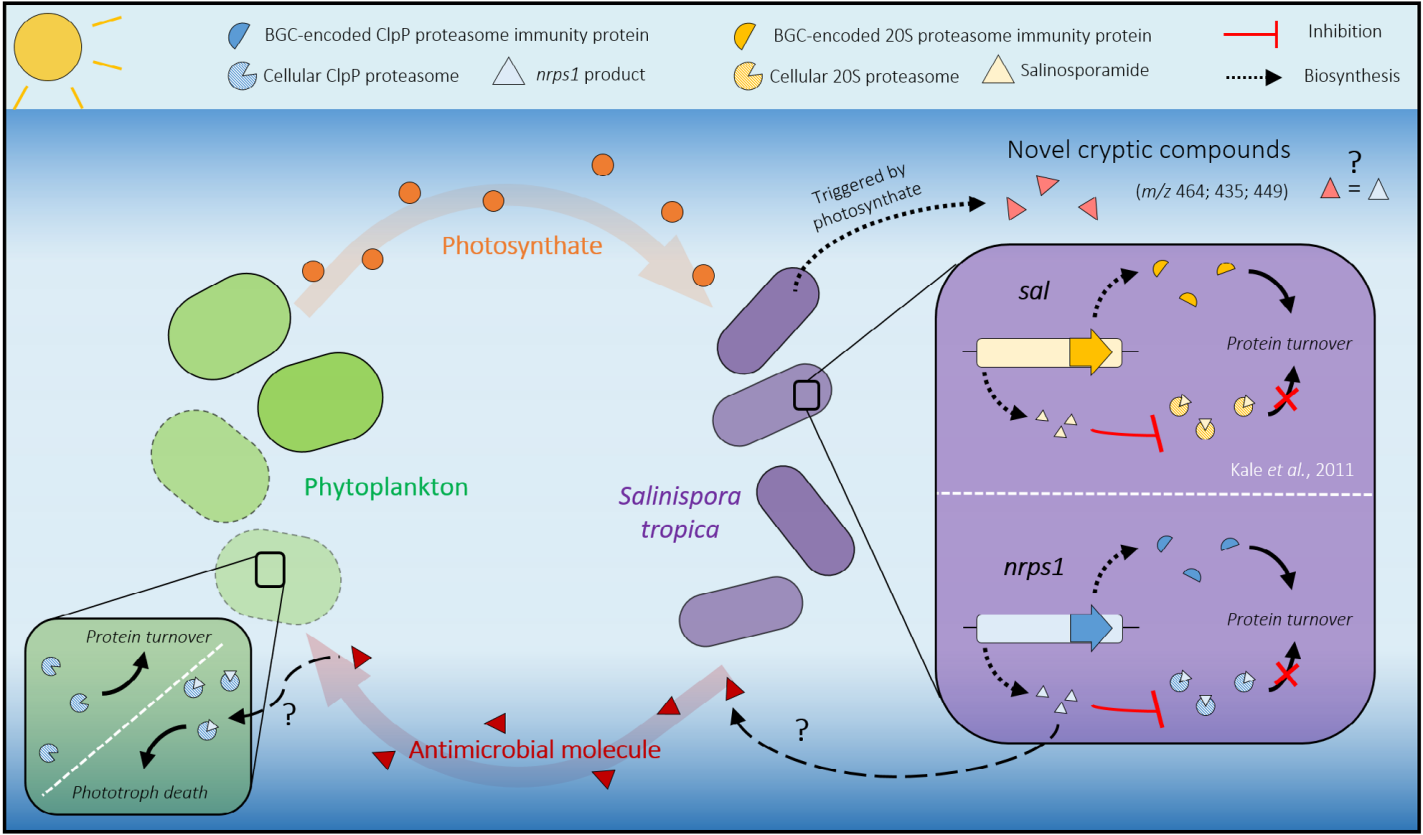
Interaction of *Salinispora tropica* with phytoplankton. Marine phototrophs release photosynthate that triggers the biosynthesis of novel cryptic metabolites in *S. tropica*. *S. tropica* produces an unknown antimicrobial molecule that kills phytoplankton. The proposed mechanism of the antimicrobial metabolite as well as the activity of the *nrps1* product are depicted (green and purple boxes, respectively). We hypothesize that the BGC *nrps1* may produce a ClpP proteasome inhibitor, to which *S. tropica* would be resistant because of an immunity protein encoded within the BGC, similarly to what is known for *sal*/salinosporamide. The *nrps1*‐encoded proteasome inhibitor could kill the phototrophs by preventing protein turnover, leading to cell death.

The proteins detected from the BGC *nrps1* are essential enzymes involved in non‐ribosomal peptide biosynthesis, *that is* A4X2Q0, a non‐ribosomal peptide synthetase (NRPS) made of a C‐A‐PCP domain; A4X2Q2, a ketoreductase domain‐containing protein; A4X2R4, an adenylation domain‐containing protein; and A4X2R5, a condensation domain‐containing protein made of C‐PCP‐TE domain. The detection of these proteins therefore strongly suggests the actual synthesis of the non‐ribosomal peptide and could well be the novel cryptic metabolites **4**, **6** and **7** detected by LC‐MS. The molecules indeed include four nitrogen atoms in their predicted molecular formulae (Table 2) and four identified A‐domains, which are responsible for the selection and activation of the amino‐acids monomers incorporated into the non‐ribosomal peptide, are encoded in the *nrps1* BGC. Interestingly, while the substrate specificity of A4X2Q0’s A‐domain is alanine, the one of A4X2R4 and the two others could not be predicted. We also show that the proteins encoded by the *nrps1* BGC were more abundantly detected with photosynthate (Fig. 4), which is consistent with our observation that the detection of the molecules is conditional to the presence of phytoplankton or their photosynthate. This correlation supports the orphan *nrps1* as a promising candidate for the production of the cryptic compounds and, potentially, the antimicrobial activity observed on co‐cultured phototrophs. Further work is however required to elucidate the structure of this series of cryptic metabolites. From this same BGC we also detected a highly abundant ATP‐dependent Clp protease proteolytic subunit (ClpP, A4X2S2) that may be providing *S*.* tropica* with self‐resistance against the *nrps1* peptides. Virtually all organisms across the tree of life have a system for targeted proteolysis for protein turnover, with most bacteria, mitochondria and chloroplasts relying on a ClpP‐type proteasome while eukaryotes, archaea and some actinobacteria typically possess the homologous 20S proteasome structure (Becker and Darwin, 2016; Snoberger *et al*., 2017). The ClpP proteasome is known to be the target for certain antibiotics, including the novel acyldepsipeptide (ADEP) class (Kirstein *et al*., 2009), and it is common to find alternative ClpP proteasomes encoded nearby the antibiotic‐producing BGC to confer resistance to the host cell (Thomy *et al*., 2019). In a similar fashion, salinosporamide A is a 20S proteasome inhibitor, to which *S*.* tropica* is resistant because of an extra copy of the proteasome beta subunit gene within the salinosporamide‐producing cluster (Kale *et al*., 2011). We can thus reasonably infer from the presence of *clpP* in the *nrps1* BGC that it is likely to produce an antibiotic targeting the ClpP proteasome, a class of antimicrobial compounds that has recently gained considerable attention as an attractive option to tackle multidrug resistant pathogens (Fig. 5; Momose and Kawada, 2016; Culp and Wright, 2017; Moreno‐Cinos *et al*., 2019). We here provide the first proteomic evidence that *S*.* tropica*’s *nrps1* is active and may produce a promising antimicrobial compound acting as a ClpP proteasome inhibitor. The synthesis of such an antibiotic would explain the antimicrobial effect of *S*.* tropica* on all marine phototrophs tested in our study as they all rely on the ClpP proteolytic machinery (Andersson *et al*., 2009; Jones *et al*., 2013; Zhao *et al*., 2018). Additional evidence, such as genetic inactivation of the *nrps1* BGC, will confirm this mechanism.

We show that the photosynthate released by primary producers influences the biosynthetic capacities of *S*.* tropica*, activating the expression of several orphan BGCs and inducing the production of novel metabolites. Our metabolomics analysis further confirmed the potential of co‐culturing for natural product discovery as we identified novel cryptic secondary metabolites, although future work is required to elucidate the structure of the new molecules. Finally, our study extends the pool of known compounds produced by the genus *Salinispora* and pioneers the use of phototrophs as a promising strategy to trigger novel natural products from marine actinobacteria. We also provide a valuable insight into the biosynthetic potential of *S*.* tropica* with our proteomic dataset, which reveals the *nrps1* BGC as a promising candidate for antibiotic production.

## Experimental procedures

### Culture conditions and cell abundance monitoring

#### Strains and growth media

Axenic marine phototrophs *Synechococcus* sp. WH7803, *Emiliania huxleyi* RCC1242 and *Phaeodactylum tricornutum* CCAP1055/1 were routinely grown in Artificial Seawater (ASW, Wilson *et al*., 1996), K‐media (Probert and Houdan, 2004), and F/2 media (Guillard, 1975

), respectively. Cultures were set up in Falcon 25 cm^2^ rectangular culture flasks with vented caps containing 20 ml of media and incubated at a constant light intensity of 10 μmol photons m^‐2^ s^‐1^, at 22°C with orbital shaking (140 rpm). The type strain *Salinispora tropica* CNB‐440^T^ was grown in marine broth (MB, Difco) and incubated at 30 °C with orbital shaking (220 rpm). The *S*.* tropica* mutants *salA* ^‐^ and *salL* ^‐^ were generously provided by the Moore Laboratory, USA (Eustáquio *et al*., 2008; Eustáquio *et al*., 2009).

#### Co‐culture setup

*S*.* tropica* cells were grown to late exponential phase in 10 ml of MB before washing them three times with sterile mineral media, as appropriate for each phototroph, and finally resuspending the washed cell pellet in 10 ml of mineral media. Axenic phototroph cells grown to late exponential phase and the washed *S*.* tropica* were co‐inoculated in fresh media to a concentration of 10% (v/v) and 20% (v/v), respectively. *S*.* tropica* cells were also washed and resuspended in a conditioned *Synechococcus* supernatant (SUPSYN), MB or ASW when required for the metabolomic and proteomic analyses. To obtain the conditioned supernatant, *Synechococcus* cultures were incubated for 35 days as described above before centrifugation (4000 *g* for 10 min at room temperature) and further filtration through 0.22 μm pore size filters to remove cells and particulate organic matter. Washed *S*.* tropica* cells were used to inoculate SUPSYN, MB or ASW and cultures were incubated at 22°C with shaking (140 rpm) and a light intensity of 10 μmol photons m^‐2^ s^‐1^. For the physically separated *Synechococcus*‐*S*.* tropica* co‐cultures using the porous filters, cells were grown in 24 mm transwell with 0.4 μm pore polycarbonate membrane inserts (Corning, New York, USA). *Synechococcus* cells were inoculated in the well to a concentration of 20% (v/v) and *S*.* tropica* in the insert to a concentration of 55% (v/v).

#### Flow cytometry

Phototroph cell abundance was monitored using their autofluorescence by flow cytometry using a LSR Fortessa Flow Cytometer (BD) instrument, and the BD FACSDiva acquisition software (BD). Cells were detected and gated using ex. 488 nm – em. 710/50 nm at voltage 370 V, and ex. 640 nm – em. filter 670/14 nm at voltage 480 V. To remove any *S*.* tropica* cell aggregates that would block the flow cell, samples were pre‐filtered through a sterile mesh with pore size of 35 μm (Corning) prior to analysis.

### Metabolomic analysis

#### Sample preparation

The culture supernatants were analysed by non‐targeted metabolomic using either raw or concentrated supernatants. Raw supernatants were collected by sampling 200 μl of 0.22 μm‐filtered culture milieu, prior to being mixed with an equal volume of HPLC‐grade methanol. For concentrating the supernatant, cells from 10 to 100 mL of cultures were removed by centrifugation (4000 *g* for 15 min) followed by a filtering step using a 0.22 μm vacuum filter bottle system (Corning). Pre‐purification of the compounds of interest from the supernatants was carried out by solid phase extraction using C18‐silica. Using a 90:10 A/B mobile phase (where A is water with 0.1% formic acid and B is methanol with 0.1% formic acid) the undesired polar molecules and salts passed through the silica while the compounds of interest were retained and later collected following elution with a 10:90 A/B mobile phase. The obtained fractions were dried under reduced pressure at 40°C (in a speed‐vac) and resuspended in 1–3 ml of 50:50 HPLC‐grade methanol/water solution. All samples were stored in snap‐seal amber glass vials (Thames Restek, Saunderton, UK) and kept at −20°C until analysis.

#### Low‐resolution LC‐MS

Metabolites present in the cultures were routinely analysed by reversed‐phase liquid chromatography. A Dionex UltiMate 3000 HPLC (ThermoScientific, Waltham, MA, USA) coupled with an amaZon SL Ion Trap MS (Bruker) was used. A Zorbax Eclipse Plus C18 column with dimensions 4.6 x 150 mm, 5 μm particle size (Agilent Technologies, Santa Clara, CA, USA) was employed for metabolite separation with a linear gradient of 95:5 A/B to 30:70 A/B over 5 min, followed by second linear gradient to 20:80 A/B over 10 min with a flow rate of 1 ml min^‐1^ (Mobile phase A: water with 0.1% formic acid, B: methanol with 0.1% formic acid). The mass spectrometer was operated in positive ion mode with a 100–1000 m*/z* scan range. The injected volume was 10 μL at a temperature of 25 °C. Data were processed with the Bruker Compass DataAnalysis software version 4.2 (Bruker, Billerica, MA, USA).

#### High‐resolution LC‐MS

To acquire molecular formulae information, samples were analysed using an Ultra‐high‐resolution MaXis II Q‐TOF mass spectrometer equipped with electrospray source coupled with Dionex 3000RS UHPLC was employed (Bruker). A reverse phase C18 column (Agilent Zorbax, 100 x 2.1 mm, 1.8 μm) and a guard column (Agilent C18, 10 x 2.1 mm, 1.8 μm) were used for separation applying a linear gradient of 95:5 A/B to 0:100 A/B over 20 min (Mobile phase A: water with 0.1% formic acid, B: acetonitrile with 0.1% formic acid). The injected volume was 2 μl, and the flow rate was 0.2 ml min^‐1^. At the beginning of each run, 7.5 μl of 10 mM of sodium formate solution was injected for internal calibration. The mass spectrometer was operated in positive ion mode with a 50–2500 m*/z* scan range. MS/MS data were acquired for the three most intense peaks in each scan.

### Proteomic analysis

#### Preparation of cellular proteome samples

Cultures were set up as described above and incubated for 5 days after which cells were collected by centrifuging 10 ml of culture at 4000 *g* for 10 min at 4°C. Cell pellets were placed on dry ice before storing at −20°C until further processing. The cell pellets were resuspended in 200 μl 1x NuPAGE lithium dodecyl sulphate (LDS) sample buffer (ThermoFischer Scientific), supplemented with 1% ß‐mercaptoethanol. Cell pellets were lysed by bead beating (2 x 45 s and 1 x 30 s at 6.0 m/s) and sonication (5 min), followed by three successive 5‐min incubations at 95°C with short vortex steps in between. Cell lysates containing all proteins were loaded on an SDS‐PAGE precast Tris‐Bis NuPAGE gel (Invitrogen), using MOPS solution (Invitrogen) as the running buffer. Protein migration in the SDS‐PAGE gel was performed for 5 min at 200 V, to allow removal of contaminants and purification of the polypeptides. The resulting gel was stained using SimplyBlue SafeStain (Invitrogen) to visualize the cellular proteome. The gel bands containing the cellular proteome were excised and stored at −20°C until further processing.

#### Trypsin in‐gel digestion and nano LC‐MS/MS analysis

Polyacrylamide gel bands were destained and standard in‐gel reduction and alkylation were performed using dithiothreitol and iodoacetamide, respectively, after which proteins were in‐gel digested overnight with 2.5 ng μl^‐1^ trypsin (Christie‐Oleza and Armengaud, 2010). The resulting peptide mixture was extracted by sonication of the gel slices in a solution of 5% formic acid in 25% acetonitrile, and finally concentrated at 40°C in a speed‐vac. For mass spectrometric analyses, peptides were resuspended in a solution of 0.05% trifluoroacetic acid in 2.5% acetonitrile prior to filtering using a 0.22 μm cellulose acetate spin column. Samples were analysed by nanoLC‐ESI‐MS/MS with an Ultimate 3000 LC system (Dionex‐LC Packings) coupled to an Orbitrap Fusion mass spectrometer (Thermo Scientific) using a 60 min LC separation on a 25 cm column and settings as previously specified (Christie‐Oleza *et al*., 2015).

#### Proteomic data analysis

Raw mass spectral files were processed for protein identification and quantification using the software MaxQuant (version 1.5.5.1; Cox and Mann, 2008) and the UniProt database of *S*.* tropica* CNB‐440 (UP000000235). Quantification and normalization of spectral counts were done using the Label‐Free Quantification (LFQ) method (Cox *et al*., 2014). Samples were matched between runs for peptide identification and other parameters were set by default. Data processing was completed using the software Perseus (version 1.5.5.3). Proteins were filtered by removing decoy and contaminants and were considered valid when present in at least two replicates for one condition. The relative abundance of each protein was calculated using protein intensities transformed to a logarithmic scale with base 2 and normalized to protein size. Variations in protein expression were assessed with a two‐sample *T*‐test, with a false discovery rate (FDR) *q* below 0.05 and a log(2) fold change above 2 (File S1). When comparing the MB and ASW conditions against SUPSYN, variations in protein expression were analysed by one‐way ANOVA (with a FDR *q*‐value below 0.05 and a log(2) fold change above 2) followed by Tukey’s HSD *post hoc* test when ANOVA indicated significant differences.

## Conflicts of interest

The authors declare that they have no conflicts of interest.

## Supporting information


**Fig. S1**. *Synechococcus* inhibition by *S*. *tropica* is not mediated by iron depletion. Monitoring of *Synechococcus* population grown axenically or in co‐culture with *S*. *tropica*, in media supplemented with 3, 10, 50 or 100 mg l‐1 Fe(III). Graph shows mean ± standard deviation of three biological replicates.
**Fig. S2**. Chemical structure of salinosporamide A and B, with their respective degradation products. Salinosporamide A (C15H20 35ClNO4; mass 313.11) hydrolyzes to form the molecule NPI‐0065 (5) (C15H21NO5; mass 295.14) or reacts with Tris to form the hypothetical molecule (1) (C19H30N2O7; mass 398.21). Salinosporamide B (C15H21NO4; mass 279.15) hydrolyzes to form the molecule (8) (C15H23NO5; mass 297.16) or reacts with Tris to form the hypothetical molecule (2) (C19H32N2O7; mass 400.22).
**Fig. S3**. Extracted Ion chromatograms of molecules 1 and 5 in the supernatant of *S. tropica* cultures in marine broth. A. Culture supernatant of *S. tropica* grown in marine broth supplemented with trizma base. B. Culture supernatant of *S. tropica* grown in marine broth. Graphs show molecules detected with a retention time between 8.8 and 11.1 minutes. In red is shown the extracted ion chromatogram for *m/z* 399 (± 0.5). In orange is shown the extracted ion chromatogram for *m/z* 296 (± 0.5).
**Fig. S4**. Extracted ion chromatograms of molecules 1, 2, 5 and 8 in the culture supernatant of *S. tropica* wild‐type (top panel), and the salinosporamide mutants *salA*‐ (middle panel) and *salL*‐ (bottom panel). The *salA*‐ strain does not produce salinosporamide A or any derivatives, while the *salL*‐ strain still produces salinosporamide B. Graphs show molecules detected with a retention time between 8.0 and 9.4 minutes.
**Fig. S5**. Monitoring of *Synechococcus* grown in axenic culture and in co‐culture with the wild‐type, *salA*‐ or *salL*‐ *S. tropica* strains. Graph shows mean of triplicates ± standard deviation.
**Fig. S6**. MS/MS fragmentation spectra of the cryptic molecules. High‐resolution LC/(+)ESI‐MS/MS spectra obtained for molecule 4 (A), 6 (B), and 7 (C).
**Fig. S7**. The cryptic molecules 4, 6 and 7 are related. Schematic of the cryptic compounds and their corresponding daughter ions generated by MS/MS. Observed *m/z* values detected by high‐resolution LC/(+)ESI‐MS and predicted chemical formulae for [M+H]+ are provided. N/A indicate chemical formulae that could not be generated by the DataAnalysis software.
**Table S1**. Molecular ions detected by LC‐MS in *S. tropica*‐*Synechococcus* co‐culture only. Table shows molecular ions detected by high‐resolution LC/(+)ESI‐MS. Peak numbering is based on HPLC retention time and colors indicate groups of related compounds. Observed *m/z* values and predicted chemical formulae for [M+H]+ are provided. Observed mass of main ions obtained after MS2 fragmentation are given.
**Table S2**. Summary of the proteomics dataset.
**Table S3**. Detected proteins from the *pks3* orphan BGC in *S. tropica* CNB‐440. Table shows protein identifiers, annotation and relative abundance (expressed as the abundance of the protein over the abundance of the total proteome normalized to 1).

**File S1**. Cellular proteome of *Salinispora tropica* CNB‐440 grown in different incubation media.
