# Supplementary material for: Phytoplankton trigger the production of cryptic metabolites in the marine actinobacterium Salinispora tropica
Source: Microb Biotechnol. 2020 Dec 5;14(1):291–306. doi: 10.1111/1751-7915.13722 (PMC7888443; doi:10.1111/1751-7915.13722)
Supplement: Supplementary file 1 — Fig. S1. Synechococcus inhibition by S. tropica is not mediated by iron depletion. Monitoring of Synechococcus population grown axenically or in co‐culture with S. tropica, in media supplemented with 3, 10, 50 or 100 mg l‐1 Fe(III). Graph shows mean ± standard deviation of three biological replicates. Fig. S2. Chemical structure of salinosporamide A and B, with their respective degradation products. Salinosporamide A (C15H20 35ClNO4; mass 313.11) hydrolyzes to form the molecule NPI‐0065 (5) (C15H21NO5; mass 295.14) or reacts with Tris to form the hypothetical molecule (1) (C19H30N2O7; mass 398.21). Salinosporamide B (C15H21NO4; mass 279.15) hydrolyzes to form the molecule (8) (C15H23NO5; mass 297.16) or reacts with Tris to form the hypothetical molecule (2) (C19H32N2O7; mass 400.22). Fig. S3. Extracted Ion chromatograms of molecules 1 and 5 in the supernatant of S. tropica cultures in marine broth. A. Culture supernatant of S. tropica grown in marine broth supplemented with trizma base. B. Culture supernatant of S. tropica grown in marine broth. Graphs show molecules detected with a retention time between 8.8 and 11.1 minutes. In red is shown the extracted ion chromatogram for m/z 399 (± 0.5). In orange is shown the extracted ion chromatogram for m/z 296 (± 0.5). Fig. S4. Extracted ion chromatograms of molecules 1, 2, 5 and 8 in the culture supernatant of S. tropica wild‐type (top panel), and the salinosporamide mutants salA‐ (middle panel) and salL‐ (bottom panel). The salA‐ strain does not produce salinosporamide A or any derivatives, while the salL‐ strain still produces salinosporamide B. Graphs show molecules detected with a retention time between 8.0 and 9.4 minutes. Fig. S5. Monitoring of Synechococcus grown in axenic culture and in co‐culture with the wild‐type, salA‐ or salL‐ S. tropica strains. Graph shows mean of triplicates ± standard deviation. Fig. S6. MS/MS fragmentation spectra of the cryptic molecules. High‐resolution LC/(+)ESI‐MS/MS spectra obtaine [file MBT2-14-291-s001.pdf]

# Phytoplankton trigger the production of cryptic metabolites in the marine actinobacterium *Salinispora tropica*.

Audam Chhun[1,#], Despoina Sousoni[1], Maria del Mar Aguiló-Ferretjans[2], Lijiang Song[3], Christophe Corre[1,3,#], Joseph A. Christie-Oleza[1,2,4,#]

[1] School of Life Sciences, University of Warwick, Coventry, UK
[2] University of the Balearic Islands, Palma, Spain
[3] Department of Chemistry, University of Warwick, Coventry, UK
[4] IMEDEA (CSIC-UIB), Esporles, Spain

[#]Corresponding authors: a.chhun@warwick.ac.uk, c.corre@warwick.ac.uk and joseph.christie@uib.eu

# Supplementary Figures

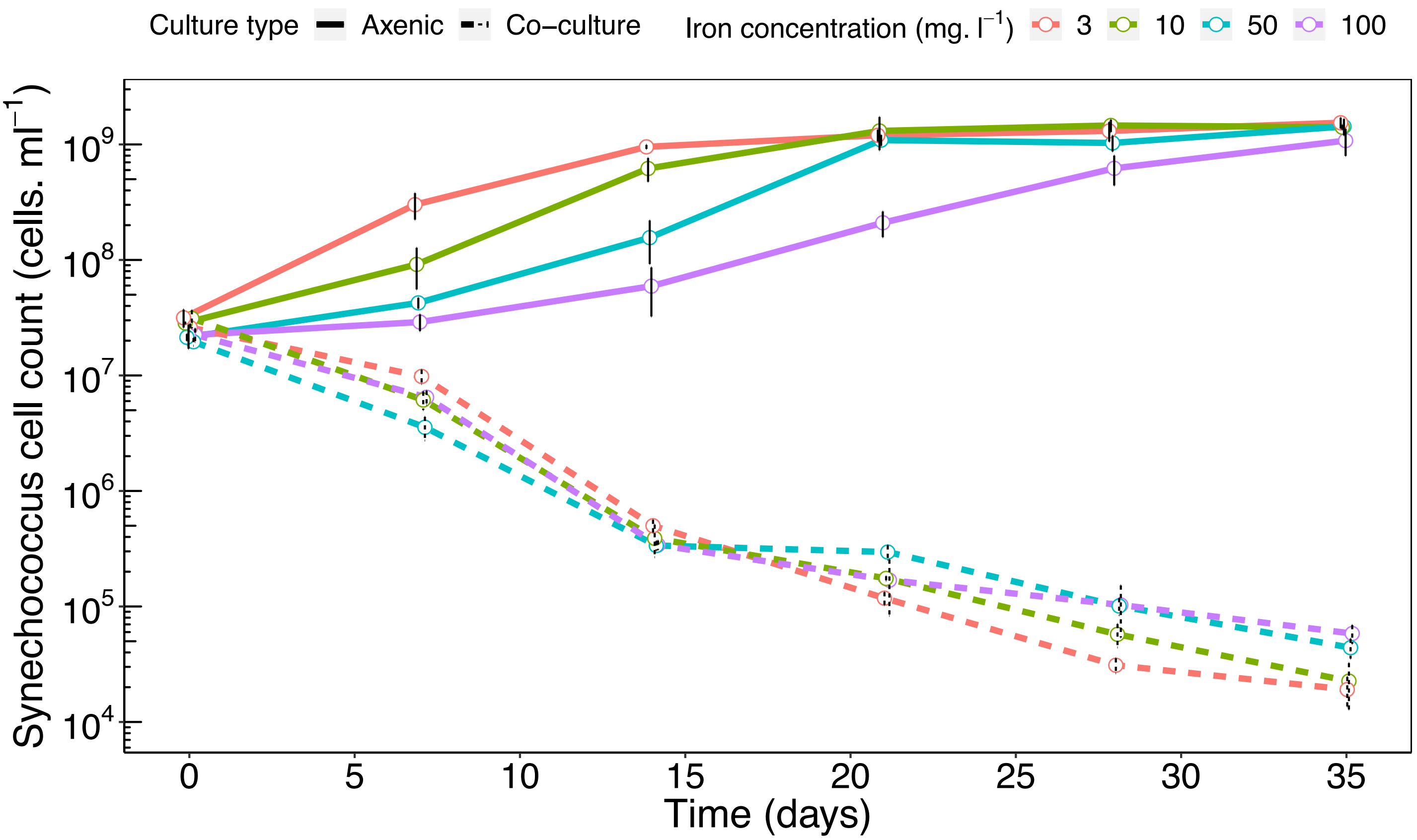


**Supplementary Figure S1 | *Synechococcus* inhibition by *S. tropica* is not mediated by iron depletion**. Monitoring of *Synechococcus* population grown axenically or in co-culture with *S. tropica*, in media supplemented with 3, 10, 50 or 100 mg $l^{-1}$ Fe(III). Graph shows mean ± standard deviation of three biological replicates.

**Supplementary Table S1 | Molecular ions detected by LC-MS in *S. tropica-Synechococcus* co-culture only.** Table shows molecular ions detected by high-resolution LC/(+)ESI-MS. Peak numbering is based on HPLC retention time and colors indicate groups of related compounds. Observed *m/z* values and predicted chemical formulae for $[M+H]^+$ are provided. Observed mass of main ions obtained after MS2 fragmentation are given.

| MS Peak | Observed *m/z* | Chemical formulae for $[M+H]^+$ (calculated *m/z*; err [ppm]) | MS/MS |
|---|---|---|---|
| 1 | 399.2135 | $[C_{19} H_{31} N_2 O_7]^+$ (399.2126; -2.4) | **296.1498**<br>271.1295<br>186.0765<br>168.0659 |
| 2 | 401.2294 | $[C_{19} H_{33} N_2 O_7]^+$ (401.2292; -2.8) | **298.1657**<br>273.1453<br>255.1348<br>152.0712 |
| 3 | 438.1701 | $[C_{28} H_{24} N O_4]^+$ (438.1700; -0.3) | 194.0817<br>177.1279 |
| 4 | 464.2509 | $[C_{22} H_{34} N_5 O_6]^+$ (464.2504; -1.2) | **276.16**<br>171.088<br>154.0615 |
| 5 | 296.1499 | $[C_{15} H_{22} N O_5]^+$ (296.1492; -2.1) | 296.1499<br>318.132<br>168.066 |
| 6 | 435.2609 | $[C_{22} H_{35} N_4 O_5]^+$ (435.2602; -1.7) | 372.229<br>**276.1599**<br>142.0979 |
| 7 | 449.2764 | $[C_{23} H_{37} N_4 O_5]^+$ (449.2758; -1.3) | 156.1135 |
| 8 | 298.1653 | $[C_{15} H_{24} N O_5]^+$ (298.1649; -1.5) | 320.1447<br>298.1654<br>170.0815 |

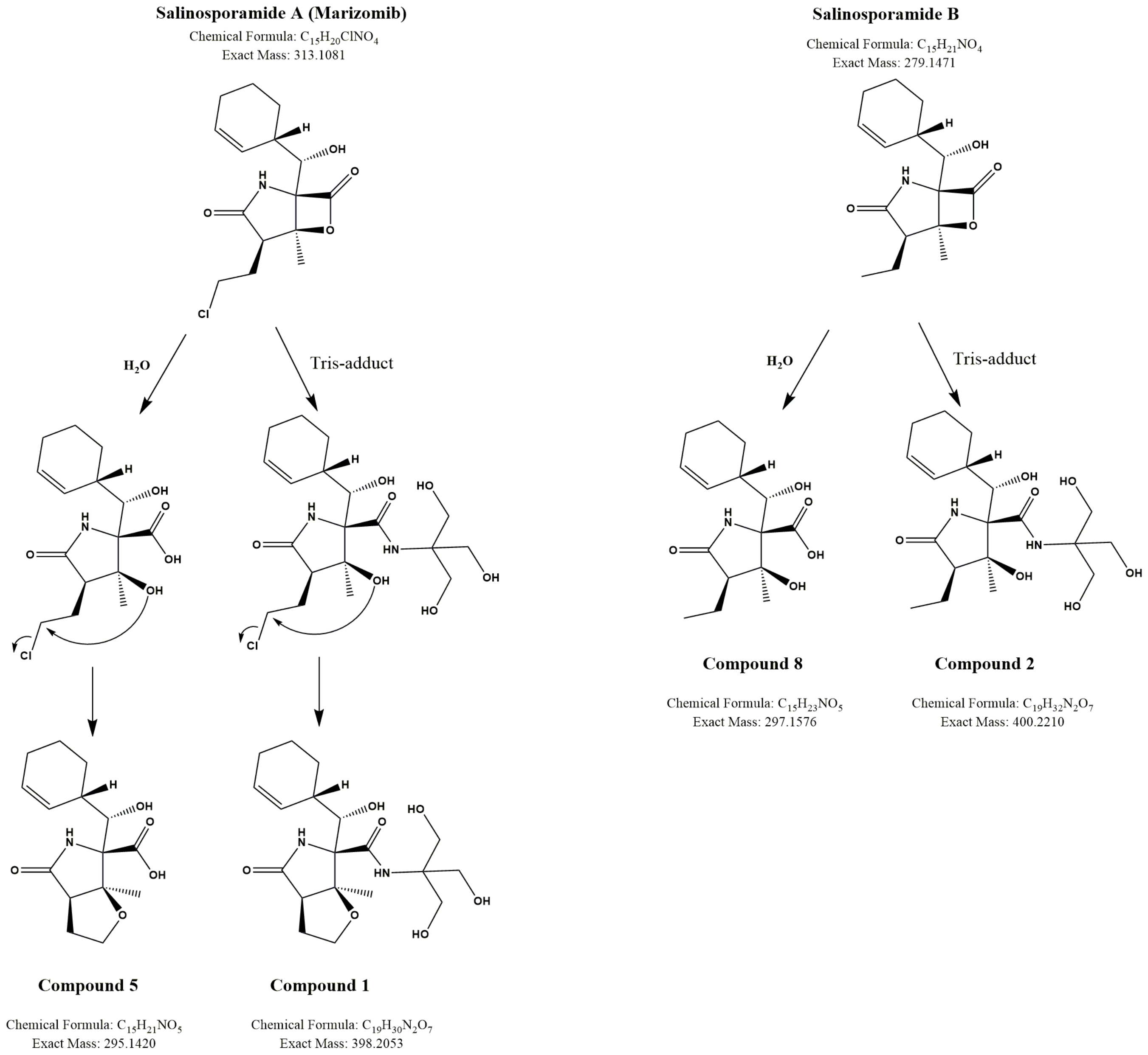


**Supplementary Figure S2 | Chemical structure of salinosporamide A and B, with their respective degradation products.** Salinosporamide A ($C_{15}H_{20}{}^{35}ClNO_4$; mass 313.11) hydrolyzes to form the molecule NPI-0065 (**5**) ($C_{15}H_{21}NO_5$; mass 295.14) or reacts with Tris to form the hypothetical molecule (**1**) ($C_{19}H_{30}N_2O_7$; mass 398.21). Salinosporamide B ($C_{15}H_{21}NO_4$; mass 279.15) hydrolyzes to form the molecule (**8**) ($C_{15}H_{23}NO_5$; mass 297.16) or reacts with Tris to form the hypothetical molecule (**2**) ($C_{19}H_{32}N_2O_7$; mass 400.22).

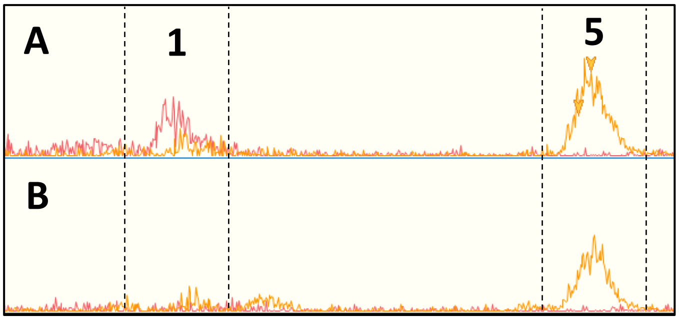


**Supplementary Figure S3 | Extracted Ion chromatograms of molecules 1 and 5 in the supernatant of *S. tropica* cultures in marine broth. A.** Culture supernatant of *S. tropica* grown in marine broth supplemented with trizma base. **B.** Culture supernatant of *S. tropica* grown in marine broth. Graphs show molecules detected with a retention time between 8.8 and 11.1 minutes. In red is shown the extracted ion chromatogram for *m/z* 399 (± 0.5). In orange is shown the extracted ion chromatogram for *m/z* 296 (± 0.5).

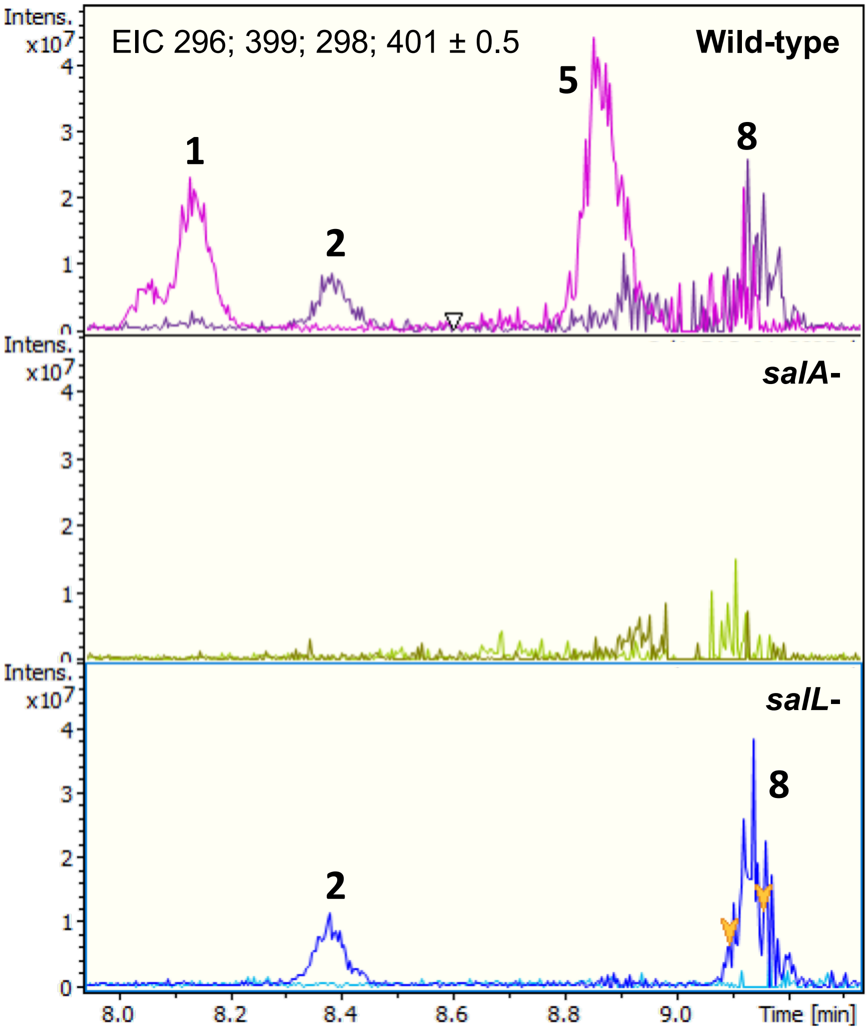


**Supplementary Figure S4 | Extracted ion chromatograms of molecules 1, 2, 5 and 8 in the culture supernatant of *S. tropica* wild-type (top panel), and the salinosporamide mutants *salA*- (middle panel) and *salL*- (bottom panel).** The *salA*- strain does not produce salinosporamide A or any derivatives, while the *salL*- strain still produces salinosporamide B. Graphs show molecules detected with a retention time between 8.0 and 9.4 minutes.

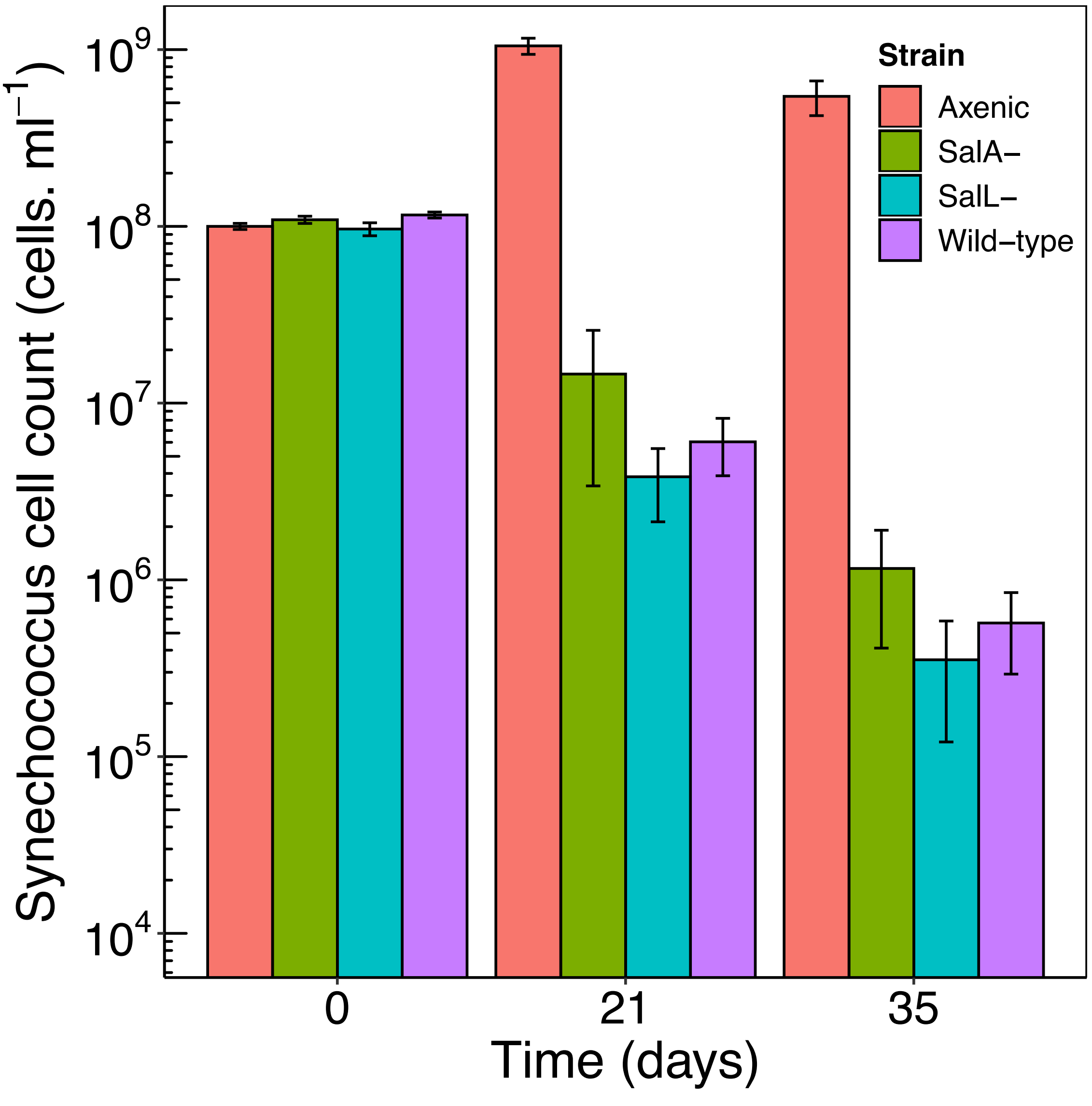


**Supplementary Figure S5 | Monitoring of *Synechococcus* grown in axenic culture and in co-culture with the wild-type, *salA*- or *salL*- *S. tropica* strains.** Graph shows mean of triplicates ± standard deviation.

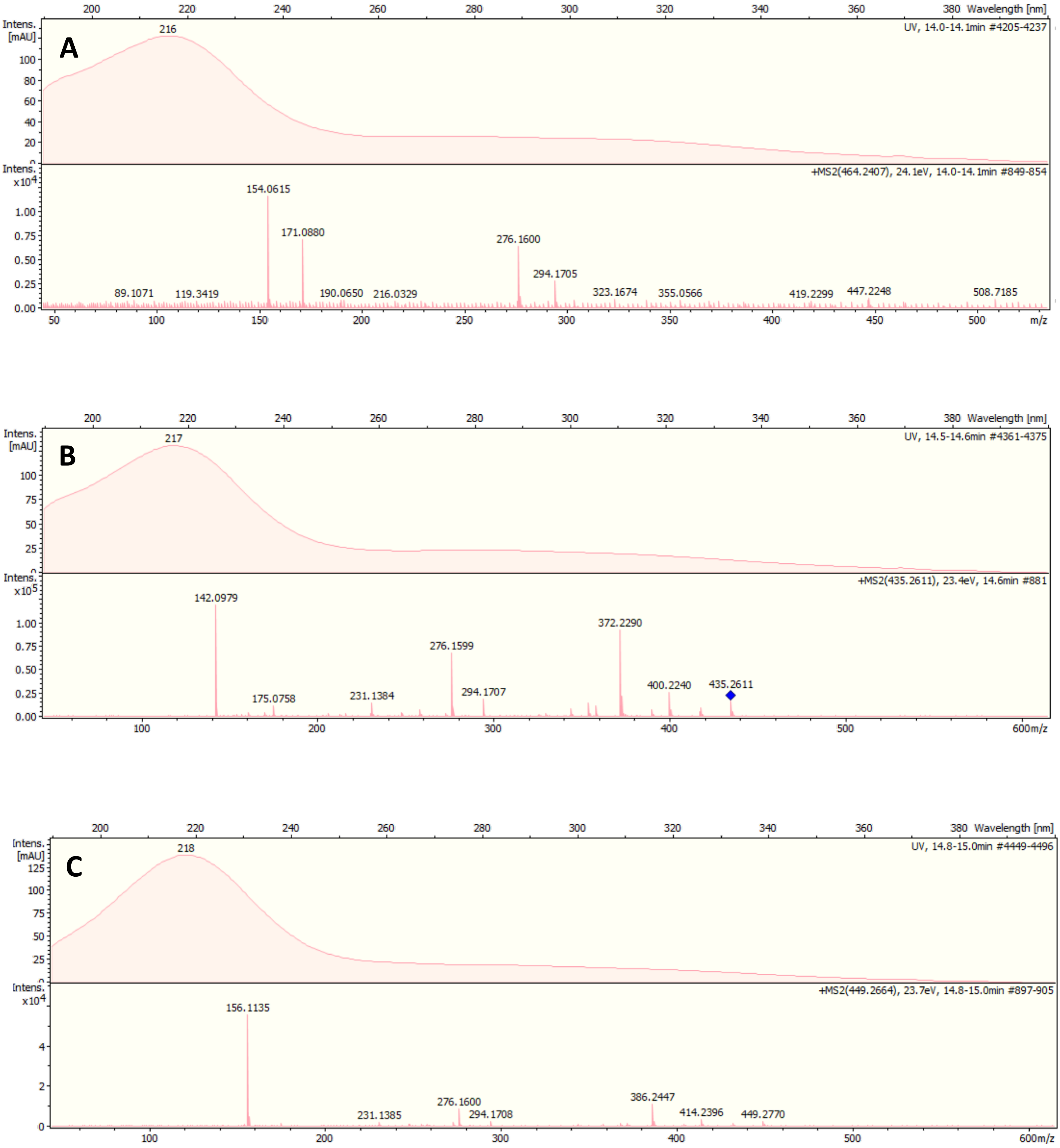


**Supplementary Figure S6 | MS/MS fragmentation spectra of the cryptic molecules.** High-resolution LC/(+)ESI-MS/MS spectra obtained for molecule **4** (**A**), **6** (**B**), and **7** (**C**).

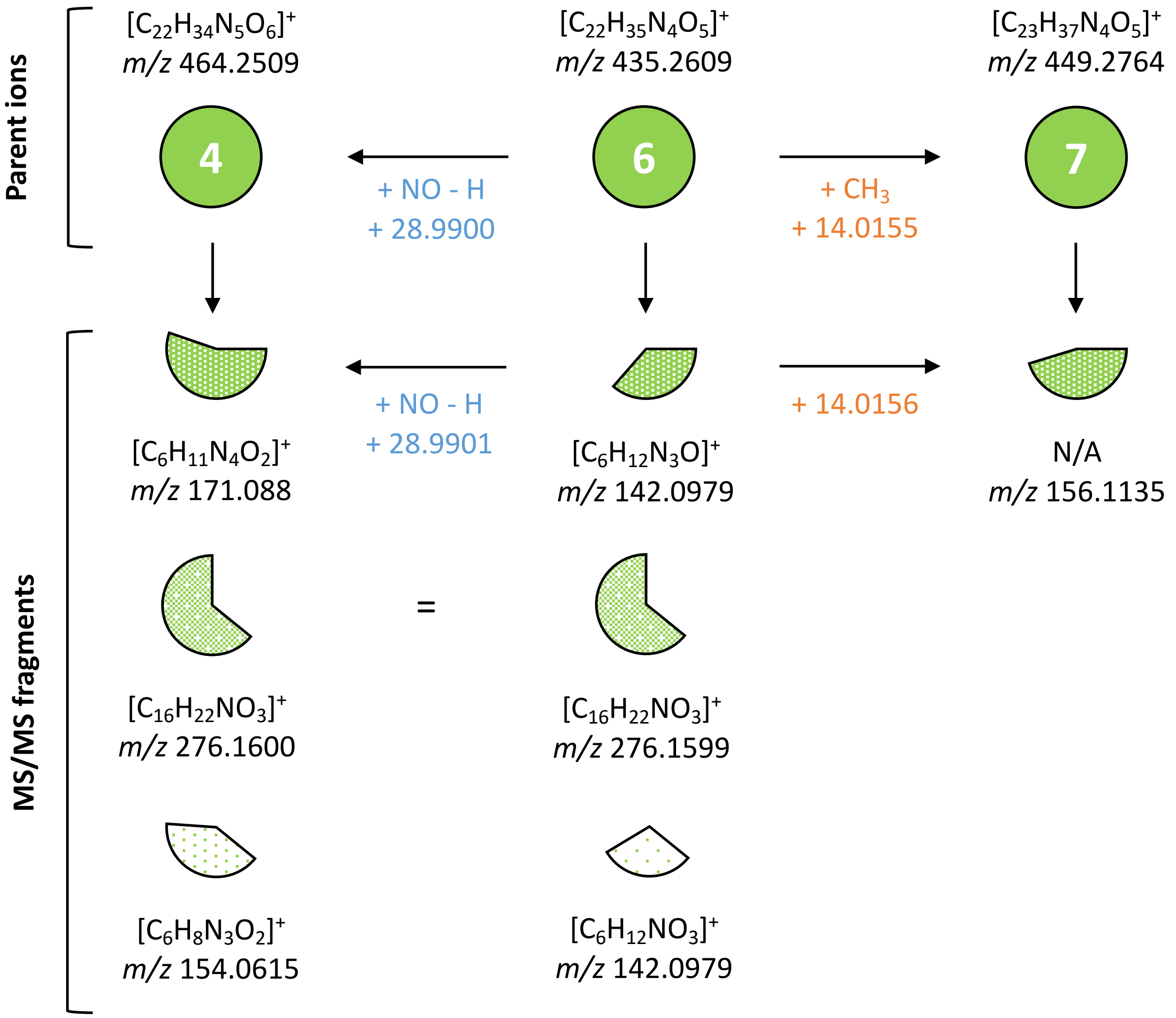


**Supplementary Figure S7 | The cryptic molecules 4, 6 and 7 are related.** Schematic of the cryptic compounds and their corresponding daughter ions generated by MS/MS. Observed *m/z* values detected by high-resolution LC/(+)ESI-MS and predicted chemical formulae for $[M+H]^+$ are provided. N/A indicate chemical formulae that could not be generated by the DataAnalysis software.

**Supplementary Table S2 | Summary of the proteomics dataset.**

| Strain studied (No. of proteins) | Growth medium | Detected proteins (% of total proteins) | Detected proteins related to BGCs (% of total detected) | Relative abundance[a] (%) |
|---|---|---|---|---|
| *S. tropica* CNB-440 (4,522) | Marine Broth (MB) | 1,869 (41.3) | 179 (9.6) | 9.6 ± 0.9 |
| | Artificial Seawater (ASW) | 1,797 (39.7) | 172 (9.6) | 11.4 ± 0.4 |
| | Phototroph supernatant (SUPSYN) | 1,831 (40.5) | 181 (9.9) | 15.0 ± 1.2 |

Significance brackets in the Relative abundance column: MB vs SUPSYN ***; ASW vs SUPSYN **.

[a] Table shows the cumulated relative abundance of the detected proteins linked to biosynthetic gene clusters, indicated as mean ± standard deviation of three biological replicates. Tukey HSD test, ** significant at $q$-value < 0.01; *** significant at $q$-value < 0.001.

**Supplementary Table S3 | Detected proteins from the *pks3* orphan BGC in *S. tropica* CNB-440.** Table shows protein identifiers, annotation and relative abundance (expressed as the abundance of the protein over the abundance of the total proteome normalized to 1).

| Protein ID | Gene name | Locus tag | Annotation | Relative abundance |
|---|---|---|---|---|
| A4X7T4 | Strop_2488 | STROP_RS12520 | DUF3050 domain-containing protein | 0.000750547 |
| A4X7T5 | Strop_2489 | STROP_RS12525 | phytanoyl-CoA dioxygenase | 0.130375917 |
| A4X7T6 | Strop_2490 | STROP_RS12530 | 4-hydroxyphenylpyruvate dioxygenase | 0.002402502 |
| A4X7T7 | Strop_2491 | STROP_RS12535 | beta-ketoacyl | 0.001818263 |
| A4X7T8 | Strop_2492 | STROP_RS12540 | acyl--CoA ligase | 0.031677582 |
| A4X7U0 | Strop_2494 | STROP_RS12550 | 3-ketoacyl-ACP synthase | 0.014597734 |
| A4X7U3 | Strop_2497 | STROP_RS12570 | long-chain fatty acid--CoA ligase | 0.034956859 |
| A4X7U4 | Strop_2498 | STROP_RS12575 | antibiotic biosynthesis monooxygenase | 0.129801561 |
| A4X7U5 | Strop_2499 | STROP_RS12580 | actinorhodin polyketide beta-ketoacyl synthase | 0.006074535 |
| A4X7U6 | Strop_2500 | STROP_RS12585 | beta-ketoacyl | 0.00502919 |
| A4X7U7 | Strop_2501 | STROP_RS12590 | cupin domain-containing protein | 0.146326909 |
| A4X7U8 | Strop_2502 | STROP_RS12595 | cyclase | 0.495965212 |
| A4X7U9 | Strop_2503 | STROP_RS12600 | acetyl-CoA carboxylase biotin carboxylase subunit | 0.006262552 |
| A4X7V0 | Strop_2504 | STROP_RS12605 | acetyl-CoA carboxylase biotin carboxyl carrier protein | 0.10444248 |
| A4X7V1 | Strop_2505 | STROP_RS12610 | acetyl-CoA carboxylase carboxyltransferase subunit alpha | 0.000773389 |
| A4X7V2 | Strop_2506 | STROP_RS12615 | TcmI family type II polyketide cyclase | 0.117717019 |
| A4X7V4 | Strop_2508 | STROP_RS12625 | TcmI family type II polyketide cyclase | 0.229200995 |
